# Fundus Examination of 23,861 Newborns by Digital Imaging in Ningbo

**DOI:** 10.1155/2021/6620412

**Published:** 2021-04-28

**Authors:** Delin Liu, Jiao Zheng, Yi Lu

**Affiliations:** Ningbo Women's and Children's Hospital, 339 Liuting Road, Ningbo 315012, China

## Abstract

**Purpose:**

To perform fundus examinations of full-term and premature infants to identify common congenital ocular diseases and determine the incidence and additional risk factors in Ningbo, China.

**Methods:**

Fundus examinations were performed on newborns between January 2017 and July 2020 in Ningbo using a RetCam3 or PanoCam LT wide-field digital imaging system. The neonates' birth weight, gestational age, gender, delivery mode, oxygen intake, and other conditions were recorded. We compared the incidence of ocular abnormalities in both full-term newborns and premature infants.

**Results:**

There were 23,861 newborns in this study comprising 12,605 (52.8%) male and 11,256 (47.2%) female infants, 20,938 full-term babies, and 2,923 premature babies. The average gestational age was 37.9 ± 5.6 weeks, and the average birth weight was 3,189 ± 417 g. Overall, we found ocular abnormalities in 6,645 (27.8%). The most common abnormality in full-term newborns was retinal hemorrhage (RH), which we found in 3,827 (18.3%) cases. Other diseases identified included familial exudative vitreoretinopathy (FEVR), retinoblastoma (RB), and congenital cataracts. The delivery method had a significant impact on the incidence of neonatal RH (*P* < 0.001). Retinopathy of prematurity (ROP) was observed in 617 newborns accounting for 21.1% of all screened premature infants. Logistic analysis showed that gestational age and birth weight were important risk factors for ROP (*P* < 0.001). For treatable diseases, such as ROP, FEVR, congenital cataract, glaucoma, and RB, early identification allows for active treatment or referral to a specialized hospital for further treatment.

**Conclusion:**

Early examination and prompt treatment of ocular disorders in newborns are important to avoid lifelong visual impairment. Eye examinations should be performed during the neonatal period and at regular follow-up visits.

## 1. Introduction

Vision is the foundation of children's intellectual and emotional development. A vision defect may influence children's intellectual development and social adaptability and potentially lead to loss of life [1, 2]. Congenital ocular diseases are the leading causes of vision loss and blindness in children and are an important issue that results in social and economic burdens globally [3]. The first 4–6 months is the crucial stage for neonatal eye development. If congenital eye diseases are not detected early, the best time for effective treatment will be missed [4], potentially leading to irreversible visual damage or a life-threatening crisis. Therefore, early screening, diagnosis, and intervention play an important role in preventing and reducing vision loss caused by congenital ocular diseases.

Congenital eye diseases include neonatal fundus hemorrhage, exudative fundus lesions, retinopathy of prematurity (ROP), retinoblastoma (RB), familial exudative vitreoretinopathy (FEVR), and other congenital developmental abnormalities [5–7]. Neonatal eye disease screening includes not only fundus photography but also a series of systematic examinations, from external to internal structures, eyelid abnormalities, periocular masses, and conjunctiva, cornea, iris, and red reflex by direct ophthalmoscope [5]. Finally, a fundus examination by Retcam3 or PanoCam LT can be performed.

Through a comparative analysis of the studies on neonatal fundus lesions in various geographic regions, the incidence and risk factors of neonatal congenital eye diseases differ by region. Related congenital eye diseases can have different high-incidence areas [8–10]. This study analyzed the results of neonatal fundus lesion screening in Ningbo, China, to clarify the incidence and risk factors of common congenital eye diseases in this area.

## 2. Methods

This study was approved by the Ethics Committee of Ningbo Women's and Children's Hospital. Before the examination, the parents of the newborns were provided with information on this study, and only those who signed written informed consent were included in this study.

### 2.1. Patients

From 1 January 2017 through 30 July 2020, we performed eye disease screening for full-term infants within 30 days of birth. For premature infants, we performed this eye examination at 32 weeks of postmenstrual age (PMA) or four weeks after birth, in accordance with the Chinese Medical Association Guild guidelines. The fundus examination was repeated weekly or, if needed, more frequently until the full vascularization of the peripheral retina was observed or until 45 weeks of postmenstrual age. Premature infants with ROP who met the treatment criteria should be treated in a timely manner and followed up closely according to their condition.

### 2.2. Fundus Examination

Before the examination, the ophthalmologist evaluated the newborn's external eyes and checked the conjunctiva, cornea, iris, and red reflex. The neonates were fasted for at least one hour before the examination. We dilated the pupils using tropicamide three times at least 30 minutes prior to examination. The newborn was placed on the examination bed, wrapped in a bag, and their head was fixed with the assistance of the examination nurse. After topical anesthesia, the eyelid was opened by a sterile eyelid speculum and we applied levofloxacin gel on the corneal surface. A few diseases, such as congenital cataracts, can be detected by red light reflection, while most diseases, such as FEVR and early RB, will still be found through screening. RetCam3 or PanoCam LT was used for 130° fundus shots. Photos were taken of the posterior pole, including the optic nerve and macula by Dr. Jiao Zheng and Dr. Yi Lu. The superior, inferior, nasal, and temporal retinal fields were imaged when possible. During the image collection process, we tried to capture the serrated edge as much as possible and focused on the lesion area. For newborns with ocular diseases, such as ROP and FEVR, further binocular funduscopy was performed by Dr. Delin Liu to confirm the diagnosis.

We used Egge's classification standard to classify RH [11] and typical ICROP classification to classify ROP [12]. For diseases requiring laser or anti-VEGF treatment, it was performed in our hospital. Untreatable diseases in our hospital, such as congenital cataracts, glaucoma, and RB, were referred to a specialized hospital.

### 2.3. Statistics

The results of examinations were analyzed using SPSS 23 (SPSS, Chicago, USA), counting data were expressed as a rate, comparison of rates in each group was expressed by *χ*^2^ test, measurement data were expressed as *x* ± *s*, and single-factor analysis was used for risk factors of retinal hemorrhage and retinopathy. Logistic regression was also used for risk factors of retinopathy. *P* < 0.05 was considered statistically significant.

## 3. Results

We collected data on 23,861 living newborns. The study involved 20,938 full-term infants and 2,923 premature infants. There were 12,605 (52.8%) male and 11,256 (47.2%) female infants in the study. The average gestational age (GA) was 37.9 ± 5.6 weeks, and the average birth weight (BW) was 3189 ± 417g. Of the newborns who underwent fundus examinations, 6,645 (27.8%) cases were found to have ocular abnormalities (Table 1).

### 3.1. Ocular Abnormalities of Full-Term Newborns and Premature Infants

**Figure 1:**
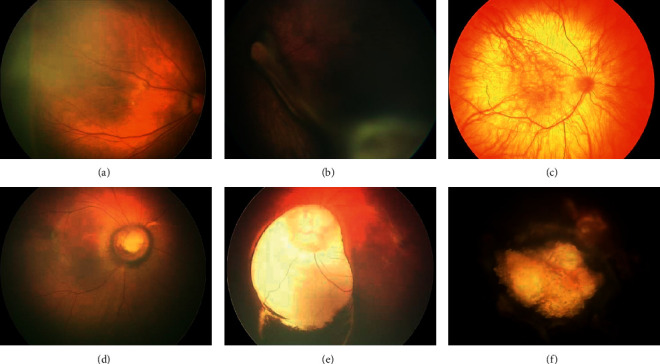
Rare ocular abnormalities were found in neonates through digital imaging. (a) Familiar exudative vitreoretinopathy. (b) Persistent fetal vasculature. (c) Albinotic fundus. (d) Morning glory syndrome. (e) Coloboma of the choroid. (f) Retinoblastoma.

We identified ocular abnormalities in both the anterior and posterior segments of the eye. Our findings included some rare ocular abnormalities (Figure 1), including congenital cataracts, glaucoma, RB, and PFV. The prevalence of ocular abnormalities was different between full-term and premature infants (*p*=0.035). The clear neonatal fundal diseases of full-term and premature neonates were also analyzed in our study (Table 2).

### 3.2. The Incidence and Related Factors of RH in Full-Term Neonates

**Figure 2:**
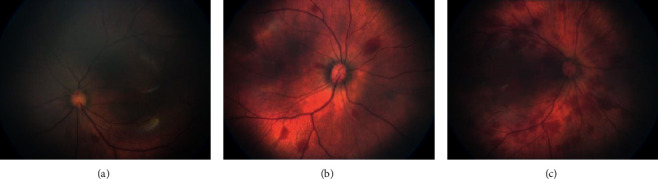
Different degrees of retinal hemorrhage were detected in newborns. (a) Grade I retinal hemorrhage. (b) Grade II retinal hemorrhage. (c) Grade III retinal hemorrhage.

The most common abnormality in full-term newborns was RH, which we found in 3827 (18.3%) cases. There were 1680 cases of grade 1 hemorrhage (43.9%), 1031 cases of grade 2 hemorrhage (26.9%), and 1116 cases of grade 3 hemorrhage (29.2%). Vaginal birth was a risk factor for RH (*p* < 0.001). Birth weight and fetal distress were also risk factors for RH (*p* < 0.001). However, gender, maternal gestational diabetes, and maternal hypertension during pregnancy had no obvious influence on the incidence of neonatal retinal hemorrhage (Table 3; *p* > 0.005). We detected different degrees of retinal hemorrhage (Figure 2).

### 3.3. The Incidence and Related Factors of ROP in Premature Infants

**Figure 3:**
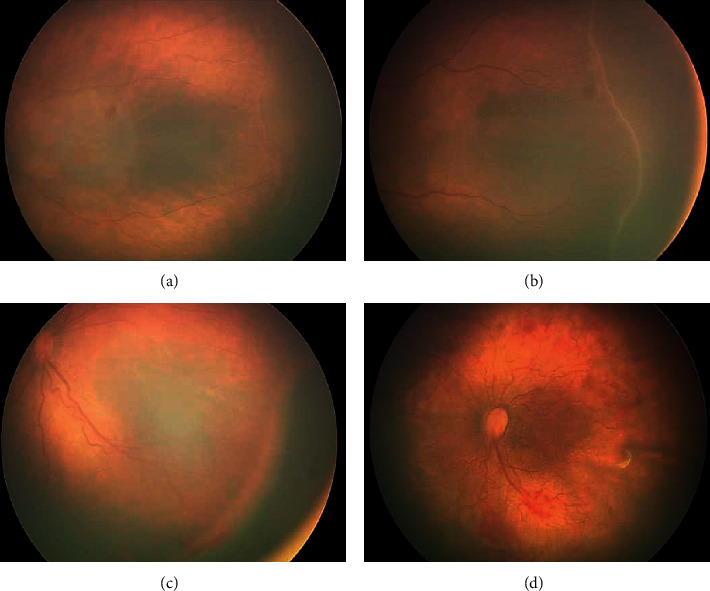
ROP found in premature newborns. (a) Grade I ROP in a newborn with gestational age of 29 weeks + 5 days. (b) Grade II ROP in a newborn with gestational age of 28 weeks + 3 days. (c) Grade III ROP in a newborn with gestational age of 27 weeks + 6 days. (d) Plus disease ROP in a newborn with gestational age of 26 weeks + 4 days.

ROP was observed in 617 newborns, accounting for 21.1% of all screened premature infants. We detected different degrees of ROP (Figure 3). Among preterm infants with different gestational ages, the difference in the incidence rate of ROP was statistically significant (*P* < 0.001). The incidence of ROP in premature infants in the 26–28+6 weeks age group was significantly higher than that of other gestational age groups. The older the gestational age, the smaller the detection prevalence of ROP, showing a significant inverse trend (Table 4). Among preterm infants with different birth weights, the difference in the rate of ROP was statistically significant (*p* < 0.001); the incidence of ROP in body weight in the below 1000g group was significantly higher than other groups. The greater the birth weight, the lower the detection rate of ROP (Table 4). Moreover, the prevalence of ROP in the human milk feeding group was significantly lower than in the formula milk feeding group (*p* < 0.001). The incidence of ROP in the oxygen inhalation group was significantly higher than that in the nonoxygen group (*p* < 0.001). Further logistic regression analysis showed that BW and GA were the most relevant risk factors (Table 5).

### 3.4. Treatment and Referral for Diseases That Require Treatment

For 93 ROP patients that required treatment, we performed anti-VEGF or laser therapy according to the International ROP Treatment Standard. For 23 FEVR patients complicated with neovascularization, retinal laser therapy was performed. For grade 1 and 2 RH, the hemorrhage was basically absorbed within 2–4 weeks. When accompanied by severe macular hemorrhage, vitamin C and vitamin K were added to the treatment, and the absorption was almost complete within eight weeks. Congenital cataracts, glaucoma, RB, and other diseases that cannot be treated in our hospital are referred to superior hospitals for treatment.

For untreatable congenital diseases, such as choroidal coloboma and morning glory syndrome, parents were informed of the prognosis of the disease and reminded of regular follow-up to prevent complications, such as retinal detachment, to avoid further vision loss caused by complications.

## 4. Discussion

With increasing emphasis on eye health in China, especially eye diseases that cause severe vision loss in children, the concepts of early and regular eye examinations and long-term follow-up have gradually been adopted by ophthalmologists, parents, and other individuals involved in healthcare. The American Association of Pediatrics (AAP) recommends the use of red reflex testing (RRT) to screen for eye diseases after birth [13]. However, RRT has poor performance in detecting posterior abnormalities such as retinoblastoma. Ming Sun [14] performed RRT and comprehensive eye examinations including RetCam fundus imaging in 7641 newborns. The proportion of abnormalities that were correctly classified by RRT was greater in anterior segment group (sensitivity = 99.6%) than in the posterior group (sensitivity = 4.1%). Most fundus diseases would be missed. Therefore, fundus examination of all newborns might be a more effective and sensitive method for early detection of ocular diseases.

Our study collected data from more than 20000 newborns in Ningbo. We have shown that 27.8% of all screened neonates were found to have ocular diseases, which was higher than previous studies [9, 15]. The most prevalent abnormality of full-term infants was RH, which accounted for 66.2% of all abnormal cases. 85.9% of RH cases were delivered by the vagina. Neonatal RH is usually associated with birth trauma and is often self-limited [16]. In the study of He Tang [9], 18198 (9.11%) abnormal cases were detected from 199 851 newborns. The most frequent abnormality was severe RH found in 12 810 cases (6.41%). The lower detection rate than in this study may be due to the fact that they included only severe RH. In their study, 87.4% were delivered by the vagina, similar to this study. The other anomalies including FEVR, ROP, and abnormal fundus pigmentation were also detected. In the study of Anand Vinekar [15], 48 of the 1021 full-term newborns had abnormal findings (4.7%). RH was the most common (52.1%). ROP-like ridge was noted in nine babies accounting for 18.8% of abnormalities and 0.9% of all babies. Compared with this study, it had a lower abnormal detection rate and a higher ROP-like ridge rate, perhaps due to differences in sample size.

ROP is a proliferative vascular disease that affects retinal blood vessel development in premature infants [17, 18]. With the continuous improvement of obstetrics and neonatal intensive care technology, the survival rate of premature infants has increased significantly, and the incidence of ROP has also increased in China [19, 20]. Most of the 617 premature infants with ROP in our study were in grade 1 or 2. Ultimately, the blood vessels in most of those infants grew into the serration after the boundary or ridge subsided. However, 39 infants received anti-VEGF treatment or fundus laser treatment, and two babies were transferred to a superior hospital for retinal surgery due to retinal detachment. In our study, low birth weight, small gestational age, and oxygen inhalation were associated with a higher incidence of ROP [21, 22]. Logistic regression analysis showed that BW and GA were the most important risk factors for ROP, while oxygen and milk feeding were not. It is possible that children on oxygen and milk feeding were associated with lower BW and GA.

In full-term infants, we found that in addition to retinal hemorrhage, the most common ocular abnormalities were FEVR and ROP-like retinopathy. FEVR is an inherited vitreoretinal disease of retinal vascular development which can result in retinal detachment and severe visual impairment [23]. In this study, it was observed that full-term infants with FVER were affected in both eyes and were of different grades; most of the retinopathy did not require intervention. The lesions improved or stabilized after 1 to 6 months of follow-up. However, 15 infants received fundus laser treatment due to neovascularization. ROP-like retinopathy occurs in full-term and near full-term infants without a family history of similar retinal changes and can potentially lead to permanent visual impairment [24]. Most of these neonatal retinopathies were resolved on follow-up.

In addition to the above-mentioned common ocular diseases, our study population had some rare ocular defects, such as morning glory syndrome and RB. Although the incidence of these abnormalities is relatively low, early detection and intervention are of great importance to the visual development of infants.

Our study had some limitations. First, we did not follow up on some children with serious RH after the absorption of the hemorrhage. Therefore, we cannot evaluate what would influence the vision of these infants in the future. Second, considering the limited treatment technology in our hospital, some newborns with rare ocular diseases were transferred to other hospitals for further treatment and were lost to follow-up. Third, we did not analyze how many severe eye diseases requiring intervention would be missed if only routine examinations were performed.

In conclusion, our study reported the fundus examination of neonates in Ningbo through a large sample, and the results we obtained were somewhat different from those reported in other regions. Early examination of ocular disorders in newborns is important. To avoid blindness, aggressive treatment or referral is required. Examination of the eyes should be performed in the newborn period and at regular follow-up visits.

## Figures and Tables

**Table 1:** Demographics of 23,861 newborns in Ningbo.

Demographics of 23,861 newborns in Ningbo	
Gender, *n* (%), male	12,605 (52.8%)
Birth weight (g), mean (SD)	3189 ± 417 g
Gestational age (weeks), mean (SD)	37.9 ± 5.6 weeks
Ocular abnormalities, *n* (%)	6645 (27.8%)

**Table 2:** Ocular abnormalities of newborns in Ningbo.

Ocular abnormalities	Full term (*n* = 20938)	Premature (*n* = 2923)	*χ* ^2^	*P*
*Anterior segment*				
Congenital anomalies of the cornea	22 (0.001)	2 (0.001)	0.343	0.558
Congenital cataract	19 (0.001)	3 (0.001)	0.039	0.843
Congenital glaucoma	13 (0.001)	3 (0.001)	0.629	0.428

*Posterior segment*				
Retinal hemorrhage	3827 (0.183)	186 (0.064)	191.796	<0.001
ROP and ROP-like retinopathy	98 (0.005)	617 (0.211)	3759.30	<0.001
Retinal exudate	1590 (0.076)	35 (0.012)	546.008	<0.001
FVER	127 (0.006)	0 (0.000)	17.824	<0.001
PFV	11 (0.001)	1 (0.000)	0.171	0.679
Coloboma of the choroid	37 (0.002)	5 (0.002)	0.005	0.946
Albinotic fundus	32 (0.002)	9 (0.003)	3.594	0.058
Morning glory syndrome	5 (0.000)	1 (0.000)	0.109	0.741
Retinoblastoma	2 (0.000)	0 (0.000)	0.279	0.597
Total	5783 (0.276)	862 (0.295)	4.467	0.035

ROP: retinopathy of prematurity; FVER: familiar exudative vitreoretinopathy; PFV: persistent fetal vasculature.

**Table 3:** Factors affecting retinal hemorrhage in full-term infants by univariate analysis.

Affecting factors		Total	*N* (%)	*χ* ^2^	*P*
Gender	Male	11,022	1996 (0.181)	0.443	0.506
	Female	9916	1831 (0.185)		

Delivery mode	Vaginal delivery	11,984	3289 (0.274)	1576.614	<0.001
	Caesarean section	8954	538 (0.060)		

Hypertension	Yes	1587	298 (0.188)	0.287	0.592
	No	19,351	3529 (0.182)		

Diabetes	Yes	2019	374 (0.185)	0.091	0.763
	No	18,919	3453 (0.182)		

Birth weight	＜2500 g	1210	181 (0.150)	16.48	<0.001
	2500–3500 g	14,895	2691 (0.181)		
	≥3500 g	4833	955 (0.198)		

Fetal distress	Yes	1873	468（0.250）	61.98	<0.001
	No	19,065	3359（0.176）		

**Table 4:** Factors affecting ROP in premature infants by univariate analysis.

Affecting factors		Total	*N* (%)	*χ* ^2^	*P*
Gender	Male	1583	338 (0.214)	0.123	0.726
	Female	1340	279 (0.208)		

Delivery mode	Vaginal delivery	612	123 (0.201)	0.475	0.491
	Caesarean section	2311	494 (0.214)		

Hypertension	Yes	321	72 (0.224)	0.378	0.539
	No	2602	545 (0.209)		

Diabetes	Yes	358	79 (0.221)	0.225	0.635
	No	2565	538 (0.210)		

Gestational age	26–28 + 6w	73	49 (0.671)	381.789	<0.001
	29–31 + 6w	819	305 (0.369)		
	32–34 + 6w	1298	257 (0.188)		
	35–36 + 6 w	733	6 (0.009)		

Birth weight	＜1000 g	69	46 (0.681)	404.664	<0.001
	1000–2000 g	1187	423 (0.356)		
	2000–3000 g	1453	147 (0.101)		
	＞3000 g	214	0 (0.000)		

Feeding methods	Human milk	1098	289 (0.263)	28.689	<0.001
	Formula	1825	328 (0.180)		

Oxygen inhalation	Yes	1921	523 (0.272)	125.913	<0.001
	No	1002	94 (0.094)		

**Table 5:** Factors affecting ROP in premature infants by logistic regression analysis.

Affecting factors	Wald	Significance	Odds ratio	95% CI
Gestational age	62.025	＜0.001	0.734	0.678–0.793
Birth weight	20.101	＜0.001	0.748	0.662–0.834
Gender	0.827	0.469	0.876	0.653–1.177
Delivery mode	0.010	0.915	1.027	0.757–1.395
Hypertension	0.125	0.756	0.937	0.652–1.345
Diabetes	0.136	0.744	0.947	0.662–1.355
Feeding methods	2.194	0.138	1.828	0.824–3.724
Oxygen inhalation	0.483	0.446	1.257	0.659–2.401

## Data Availability

The data used to support the findings of this study are available from the corresponding author upon request.
